# Isolationof PASN from Argentine Squid Carcass By-Products Enhances Proliferation and Repair of hACs and PC12 In Vitro via Antioxidant Activity

**DOI:** 10.3390/foods15111844

**Published:** 2026-05-23

**Authors:** Haoze Yang, Tianming Wang, Yaqi Kong, Qian Yao, Huiying Wang, Bailin Li, Jeevithan Elango, Wenhui Wu

**Affiliations:** 1 Department of Marine Bio-Pharmacology, College of Food Science and Technology, Shanghai Ocean University, Shanghai 201306, China; m230351143@st.shou.edu.cn (H.Y.); wtm4411@163.com (T.W.); m230351113@st.shou.edu.cn (Y.K.); m240451305@st.shou.edu.cn (Q.Y.); m240401149@st.shou.edu.cn (H.W.); blli@shou.edu.cn (B.L.); 2 Putuo Sub-Center of International Joint Research Center for Marine Biological Sciences, Zhoushan 316104, China; 3 Department of Biomaterials Engineering, Faculty of Health Sciences, UCAM—Universidad Católica San Antonio de Murcia, Campus de los Jerónimos, 135, Guadalupe, 30107 Murcia, Spain; 4 Center of Molecular Medicine and Diagnostics (COMManD), Department of Biochemistry, Saveetha Institute of Medical and Technical Sciences, Saveetha Dental College and Hospitals, Saveetha University, Chennai 600077, India; 5 Marine Biomedical Science and Technology Innovation Platform of Lin-Gang Special Area, Shanghai 201306, China

**Keywords:** PASN, Argentine squid, marine by-products, antioxidant peptide, cytoprotective effect

## Abstract

Marine by-products represent a promising source of bioactive peptides. This study aimed to isolate and characterize a low-molecular-weight peptide fraction with antioxidant activity from Argentine shortfin squid carcass by-products, and to evaluate in vitro its cytocompatibility and protective effects against corticosterone (CORT)-induced oxidative injury in rat adrenal pheochromocytoma (PC12) cells and human astrocyte (hACs) cells. Argentine squid antioxidant peptide (PASN) was obtained by size-exclusion chromatography and fractionation-based screening. PASN exhibited the strongest overall free-radical-scavenging activity and consisted predominantly of components below 1 kDa (211.73–1013.48 Da). Spectroscopic analyses indicated that enzymatic hydrolysis transformed its structure from a rigid triple-helix conformation to a more flexible conformation dominated by β-turns (50.78%) and random coils (17.38%). In addition, thermogravimetric analysis confirmed its excellent thermal stability, with an onset decomposition temperature as high as 244.81 °C, supporting its potential applicability in high-temperature food-processing matrices. In vitro assays demonstrated that PASN exhibited high biocompatibility and promoted proliferation of both PC12 cells and hACs, while significantly improving cell viability under CORT challenge. PASN also reduced lactate dehydrogenase (LDH) leakage (hACs: 38.31%; PC12: 31.17%) in both cell models and restored total superoxide dismutase (T-SOD) activity (hACs: 69.46%, PC12: 66.40%). Immunofluorescence further revealed that PASN rescued the expression of brain-derived neurotrophic factor (BDNF) (hACs: 35.23%, PC12: 12.50%) and glutamate decarboxylase (GAD1/2) (hACs: 102.66%, PC12: 31.31%), key markers associated with synaptic plasticity and GABAergic sleep regulation. Collectively, PASN is a thermally stable squid-derived peptide fraction that exerts antioxidant and cytoprotective effects in neural cell models in vitro and represents a promising sustainable candidate for nutraceutical development.

## 1. Introduction

Sleep disorders and neurodegenerative diseases have emerged as prevalent global health concerns, affecting a substantial proportion of the population and imposing a severe burden on individuals and society. Authoritative data indicate that neurodegenerative diseases affect approximately 40 to 50 million individuals globally. Sleep disorders are extremely common in this patient population. For example, the prevalence of sleep disorders among those with Parkinson’s disease (PD) can be as high as 60% to 90% [1,2]. Insomnia, memory impairment, and mood disorders closely associated with these conditions are typically linked to hypothalamic–pituitary–adrenal (HPA) axis dysfunction [3], which triggers excessive secretion of CORT, a pivotal glucocorticoid [4]. Elevated CORT levels disrupt the balance between reactive oxygen species (ROS) generation and antioxidant defense systems, thereby inducing neuronal oxidative stress, cellular damage, impaired neurotransmission, and eventual neurological dysfunction [5,6]. Oxidative stress has been identified as the central mechanism underlying corticosteroid-induced neurotoxicity [7], rendering antioxidant strategies a highly promising therapeutic intervention.

Marine organisms represent a rich reservoir of bioactive peptides exhibiting diverse physiological functions, particularly potent antioxidant and anti-inflammatory properties [8,9]. However, the literature on the neuroprotective properties of marine bioactive peptides remains limited, with most studies focusing on peptides from other sources [10]. Therefore, we utilized Argentine shortfin squid by-products as the starting material, selected low-molecular-weight peptides, and validated their effects using a CORT-injured cell model. Squid, a globally distributed and abundant cephalopod, has attracted substantial research attention for its high protein content; meanwhile, the underutilization of its processing by-products exacerbates environmental waste problems [11]. Prior research has confirmed that enzymatic proteolytic hydrolysates from a range of squid species display remarkable antioxidant activities [12,13]. For example, enzymatically prepared squid muscle hydrolysate has been previously documented to efficiently scavenge free radicals and protect cells against oxidative damage [14,15]. However, studies focusing on the isolation of low-molecular-weight antioxidant peptides from Argentine squid carcasses and their potential neuroprotective effects against corticosterone-induced oxidative stress remain scarce.

Bioactive peptides with a molecular weight below 1000 Da are highly preferred owing to their higher bioavailability, faster absorption, and stronger biological activity relative to large-molecular-weight proteins [16]. Enzymatic hydrolysis, especially using a two-enzyme system, is an efficient approach to producing such peptides by cleaving complex proteins into small functional fragments [17,18]. Studies have demonstrated that two-enzyme hydrolysis can significantly improve peptide yield and antioxidant capacity by generating a broader spectrum of peptide fragments [19].

Neuroprotective peptides have been shown to alleviate CORT-induced cellular damage by modulating antioxidant enzymes, reducing LDH release, and regulating the expression of neurotrophic factors and neurotransmitter-associated proteins [20,21]. BDNF is critical for neuronal survival, differentiation, and synaptic plasticity, and its downregulation is closely implicated in sleep disturbances and cognitive dysfunction [22]. GAD1/2 is a key enzyme responsible for synthesizing γ-aminobutyric acid (GABA), an inhibitory neurotransmitter that modulates the sleep–wake cycle. Reduced GAD1/2 expression leads to GABA insufficiency and subsequent sleep disturbances [23,24]. Therefore, restoring the expression of BDNF and GAD1/2 represents a pivotal therapeutic target for developing interventions against CORT-induced neurological disorders.

Argentine squid is an abundant cephalopod with protein-rich processing by-products, providing a sustainable substrate for producing bioactive peptides [9]. PC12 cells are widely used as a neuron-like model for glucocorticoid (CORT)-induced oxidative injury [25,26], whereas astrocytes are central regulators of redox homeostasis and neuronal support [27]. Therefore, hACs complement PC12 to evaluate cytoprotection in two relevant neural cell contexts.

Despite the growing research interest in marine bioactive peptides, systematic investigations into the neuroprotective effects of Argentine squid-derived peptides against cortisol-induced oxidative stress in neurons remain relatively scarce. Astrocyte-derived cells play a critical role in maintaining neuronal homeostasis, regulating synaptic transmission, and modulating antioxidant defense, rendering them a vital target for neuroprotective strategies [27].

This study aimed to isolate and purify a low-molecular-weight antioxidant peptide (PASN) from Argentine squid protein hydrolysates, followed by comprehensive structural characterization and an in-depth evaluation of its cytoprotective effects in corticosterone-induced injury models of neuron-related cells. The neuroprotective effects of PASN against CORT-induced oxidative stress in PC12 and hACs cells were evaluated by assessing cell viability, LDH release, T-SOD activity, and the expression levels of BDNF and GAD1/2. We hypothesized that PASN could alleviate CORT-induced oxidative injury by reducing oxidative stress-associated damage and restoring antioxidant defenses in neural cells. These findings provide novel insights into the potential application of Argentine squid-derived peptides as natural antioxidants and functional food ingredients for ameliorating sleep disorders and neurodegenerative diseases.

## 2. Materials and Methods

### 2.1. Chemicals and Reagents

Argentine squid was donated by Zhejiang Zhoushan Haixin Food Co., Ltd. (Zhoushan, China). Protease (BR, 64006837XW01906859101, 4 °C), acetic acid (AR, S27225500MLR, RT), and other analytical reagents were purchased from Sinopharm Chemical Reagent Co., Ltd. (Shanghai, China). Lactate dehydrogenase (LDH) (A020-2, 4 °C) and superoxide dismutase (SOD) (A001-1, 4 °C) detection kits were supplied by Nanjing Jiancheng Bioengineering Institute. (Nanjing, China). BDNF Antibody (DF6387, −20 °C), GAD1/2 Antibody (AF0711, −20 °C), and Goat Anti-Rabbit IgG (H + L) Alexa Fluor 488-conjugated (S0018, −20 °C) were obtained from Jiangsu Qinke Biological Research Center Co., Ltd. (Changzhou, China). Astrocyte medium (AM 1801, 4 °C) was purchased from ScienCell Research Laboratories (Carlsbad, CA, USA), and high-glucose DMEM (FH-D01, 4 °C) was purchased from Shanghai Fuheng Biotechnology Co., Ltd. (Shanghai, China) Unless otherwise specified, all reagents used in this study were of analytical grade.

### 2.2. Preparation of Squid Protein Peptide from Argentina

**Figure 1:**
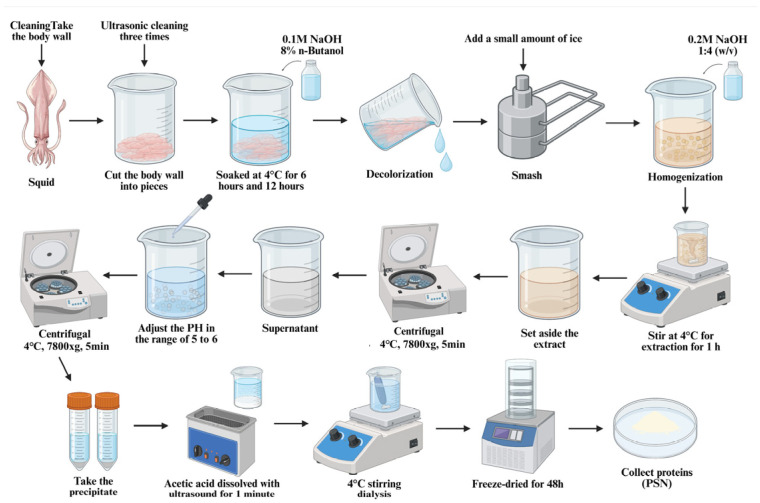
Flowchart of PSN Preparation.

After thawing, the Argentine squid was processed with the carcass retained. Based on 100 g of processed squid mantle as an example, the carcass was thoroughly rinsed with tap water, minced, and ultrasonically cleaned in ultrapure water with 1500 mL (administered in three aliquots) for 1 min (CS-500XDS, Laipute Scientific Instruments, Beijing, China). The minced samples were immersed in 0.1 M NaOH solution at a ratio of 1:5 (*w*/*v*), soaked at 4 °C for 6 h, with the solution changed once midway (consuming approximately 4.0 g of NaOH), followed by three rinses with ultrapure water. Subsequent degreasing was performed using 8% n-butanol for 12 h; the samples were then rinsed three times and reserved. The treated carcass was homogenized (A6, Shanghai Ouhé Machinery Equipment, Shanghai, China) into a paste with ice water. The homogenate was mixed with 4 volumes of 0.2 M NaOH, stirred uniformly, and extracted for 1 h. After centrifugation (Himac CR 21G, Hitachi Koki, Tokyo, Japan), the supernatant was collected, and sulfuric acid was added dropwise under stirring to reach the isoelectric point. The precipitate was harvested by centrifugation (7800× *g*, 10,000 rpm), redissolved in acetic acid solution, and ultrasonicated for 1 min. The supernatant was collected, dialyzed (MWCO = 200 Da), and gradually replaced from acetic acid to ultrapure water. Finally, the sample was lyophilized (HXLG-18-50B, Huxi Industrial, Shanghai, China) to obtain Argentine squid protein (PSN), as shown in Figure 1. All the experiments were performed in triplicate.

#### Process Yield and Mass Balance

To evaluate the feasibility of industrial production, we calculated the yields of the key processing steps. Briefly, the squid mantle by-products (taking 100 g as a representative sample) were thawed and trimmed, and their wet weight was recorded. Subsequently, the PSN was extracted via acid-solubilization and alkaline-precipitation, and the wet weight was measured to determine the process yield of PSN. The dry matter content of PSN was obtained following freeze-drying. The yields of Argentine squid peptides (PPSN) (obtained via enzymatic hydrolysis) and PASN (obtained via isolation) were calculated based on their respective freeze-dried weights. Detailed yields are provided in Supplementary Table S1. A simplified mass balance table, which includes the consumption of ultrapure water during washing/homogenization and the dosage of NaOH during alkaline immersion/extraction, is summarized in Supplementary Table S2.

### 2.3. Separation and Purification of PPSN by Sephadex Gel Chromatography

**Figure 2:**
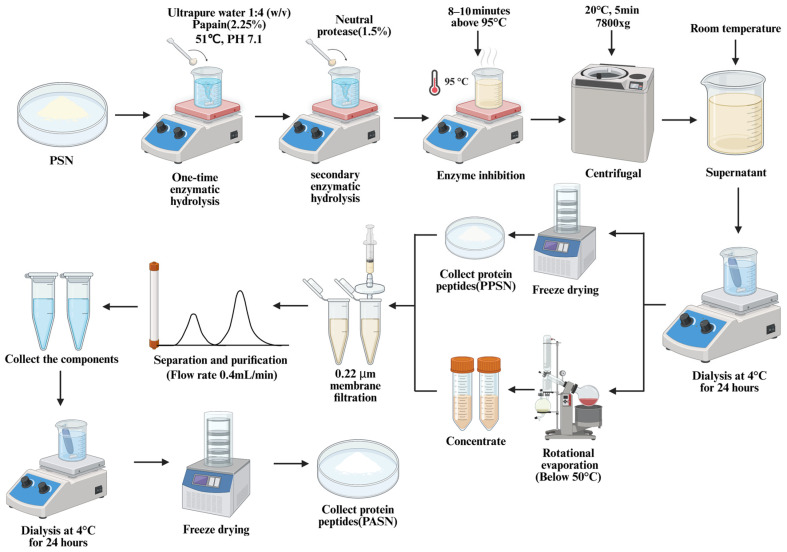
Flowchart of PPSN and PASN Preparation.

PPSN was prepared by enzymatic hydrolysis (DF-101S, Lichen Scientific Instrument, Hangzhou, China) of PSN under optimized conditions: enzymatic pH 6.8, temperature 51.2 °C, total enzyme concentration 3.75%, papain: neutral protease ratio = 3:2, and sequential hydrolysis for 3 h (papain) and 4 h (neutral protease). Detailed preparation procedures are provided in the Supplementary Materials. Sephadex G-25 was slowly packed into a chromatography column (Medium; 3 cm × 70 cm, bed volume 135 mL) and allowed to settle overnight. The column was equilibrated with ultrapure water at 25 °C until a stable baseline was achieved. For separation, a quantitative PPSN solution (3 mL, 20 mg/mL) was loaded onto the column and eluted at a flow rate of 0.4 mL/min. The elution profile was monitored at 220 nm using a UV detector (HD-3, Huxi Analytical Instrument Factory, Shanghai, China). Fractions were collected every 10 min and pooled according to the elution peaks. The fractions corresponding to the major peaks were collected, dialyzed, lyophilized, and stored for further use, as shown in Figure 2.

### 2.4. Antioxidant Capacity

PPSN, purified fractions I, II, III, and V_C_, were dissolved in ultrapure water to prepare solutions at concentrations ranging from 1 to 5 mg/mL. The DPPH [28,29], hydroxyl [28], and ABTS [30,31] radical scavenging activities were determined, with V_C_ serving as the positive control. The assays were performed according to standard spectrophotometric methods. Briefly, various concentrations (1–5 mg/mL) of the samples were mixed with the respective radical solutions. After incubation in the dark at room temperature for a specified period, the absorbance was measured. The radical scavenging activity was calculated based on the absorbance reduction compared to the control groups. Detailed procedures are provided in the Supplementary Materials.

To ensure the reliability of the antioxidant assays. The intra-day and inter-day precision were evaluated by calculating the relative standard deviation (RSD) of replicate measurements, which was found to be within 5%. All the experiments were performed in triplicate.

### 2.5. Ultraviolet-Visible (UV-Vis) Absorption Spectrum

The purity of the collagen was evaluated by determining its maximum absorption peak using a UV spectrophotometer (UV-3000PC, Mapada Instruments, Shanghai, China). PPSN and PASN were dissolved in ultrapure water to prepare 0.5 mg/mL solutions. Aliquots of 3.5 mL were transferred into clean quartz cuvettes and scanned in the range of 200–400 nm with a scanning speed of 1 nm/s and an interval of 1 nm [32]. Ultrapure water was used as a blank control.

### 2.6. Circular Dichroism (CD) Spectra

The circular dichroism spectra of the target protein peptides were recorded using a CD spectrometer (Applied Photophysics Ltd., Leatherhead, UK). During the test, peptide samples were dissolved in deionized water to a concentration of 1 mg/mL and filtered through a 0.22 μm microporous membrane. Scanning was performed at room temperature within the wavelength range of 190–260 nm. The resulting spectra were subjected to background subtraction and smoothing to characterize the secondary structure composition of the peptides in solution [33]. CD spectroscopy was used as a comparative tool to assess ensemble-averaged conformational features of PASN in aqueous solution, complementary to the solid-state information obtained by FTIR.

### 2.7. Fourier Transform Infrared Spectroscopy (FTIR)

FTIR spectra (Thermo Nicolet iS20, Thermo Fisher Scientific, Waltham, MA, USA) were recorded with air as the initial background. Approximately 2 mg of each sample (PSN and PASN) was placed on the sample plate. The scanning parameters were set as follows: resolution of 4 cm^−1^, 32 scans, and a wavenumber range of 4000–400 cm^−1^ [34]. The secondary structure was analyzed using OMNIC and PeakFit v4.12 software [35].

### 2.8. Thermogravimetric Analysis (TGA)

The thermal stability of PASN was evaluated using a thermogravimetric analyzer (TGA 550, TA Instruments, New Castle, DE, USA) [36]. Independent experiments were performed in triplicate (*n* = 3). Approximately 5 mg of PASN was accurately weighed and uniformly placed in an alumina crucible. Under a nitrogen atmosphere (purity ≥ 99.9%, flow rate: 40 mL/min), the sample was heated from 30 °C to 500 °C at a heating rate of 10 °C/min. An empty alumina crucible was used as the reference. Derivative thermogravimetric (DTG) curves were obtained by differentiating the TGA mass-loss profiles. The selected range of 30–500 °C was chosen to fully characterize the thermal decomposition behavior, capturing moisture loss, polymer backbone degradation, and residual char formation. This range is particularly relevant as it exceeds standard food processing temperatures, confirming the physical–chemical robustness of PASN for potential downstream applications in functional foods or pharmaceutical formulations [37,38].

### 2.9. Cultivation of hACs and PC12 Cells

Rat adrenal pheochromocytoma cells (PC12, Shanghai Fuheng, Shanghai, China) and human astrocyte cells (hACs, Zhejiang Mason, Huzhou, China) were cultured in high-glucose DMEM (Shanghai Fuheng Biotechnology Co., Ltd., Shanghai, China) and astrocyte medium (AM 1801; ScienCell Research Laboratories, Carlsbad, CA, USA), respectively. All the cells were maintained at 37 °C with 5% CO_2_, and the medium was refreshed every 48 h. When the cell confluency reached 80–90%, subcultivation was performed using 0.25% trypsin-EDTA (Shanghai Qida Biotechnology Co., Ltd., Shanghai, China).

### 2.10. Assessment of Cell Viability in hACs and PC12 Cells

hACs and PC12 cells were seeded in 96-well plates at a density of 1 × 10^4^ cells/well and cultured for 24 h (hACs) and 12 h (PC12 cells), respectively. Subsequently, different concentrations of PASN were added, and the cells were incubated for another 24 h. CCK-8 reagent (Omer Biotechnology, Shanghai, China) diluted in basal medium (1:10) was added to each well. The plates were kept in the dark for 3 h (hACs) and 1 h (PC12 cells), and the absorbance at 450 nm was measured to calculate cell viability. For the damage model assay, hACs and PC12 cells were seeded and cultured as described above. Corticosterone (Shanghai Yuanye Biotechnology, Shanghai, China), together with different concentrations of PASN, was added, and the cells were incubated for 24 h. The CCK-8 procedure was repeated as mentioned, and the absorbance at 450 nm was determined to evaluate cell viability.

### 2.11. ELISA Detection of LDH and SOD

hACs and PC12 cells were seeded in 12-well plates at a density of 7.5 × 10^4^ cells per well and cultured for 24 h (hACs) and 12 h (PC12 cells), respectively. The culture medium and cell lysates were collected. The LDH activity and T-SOD content were determined according to the manufacturer’s instructions. The assay sensitivity and linear detection range were 5–122.1 U/mL for T-SOD and 9–5000 U/L for LDH. The intra-assay and inter-assay coefficients of variation (CV) were 1.7% and 3.52% for T-SOD and 2.7% and 4.95% for LDH, respectively.

### 2.12. Immunofluorescence Detection of BDNF and GAD Expression Levels in Cells

hACs and PC12 cells were seeded in 24-well plates at a density of 4 × 10^4^ cells per well and cultured for 24 h (hACs) and 12 h (PC12), respectively. CORT (388.7 μM for PC12 and 798.3 μM for hACs), together with PASN (0.03125–1 mg/mL), was added, and the cells were incubated for another 24 h. The medium was aspirated, and the cells were washed three times with PBS. The cells were fixed with cold methanol at −20 °C for 30 min, followed by three PBS washes. Permeabilization was performed with 0.1% Triton X-100 for 15 min. Blocking solution (Shanghai Biocytogen Biotechnology Co., Ltd., Shanghai, China) was applied at room temperature for 10 min. After removal of the blocking solution, 150 μL of diluted primary antibody (BDNF, 1:200; GAD1/2, 1:200) was added to each well, and the cells were incubated at 4 °C overnight. The primary antibody was aspirated, and the wells were washed three times with PBST. Then, 150 μL of secondary antibody (Alexa Fluor 488/594 conjugated, 1:500) was added, and the cells were incubated at 37 °C in the dark for 1 h. After three PBST washes, the cells were stained with Hoechst 33258 (Beijing Solabio Technology Co., Ltd., Beijing, China) for 5 min at room temperature. The stain was removed, the wells were rinsed three times with PBS, and 200 μL of PBS was added to maintain hydration. Fluorescent images were captured using an inverted fluorescence microscope (CKX53, Olympus, Tokyo, Japan) equipped with a digital camera.

### 2.13. Statistical Analysis

All experiments were performed in triplicate with consistent results. Data are presented as mean ± standard deviation (SD) unless otherwise specified. Statistical analysis was conducted by one-way analysis of variance (one-way ANOVA) using GraphPad Prism 10 (GraphPad Software, La Jolla, CA, USA) and Origin 2024 (OriginLab Corporation, Northampton, MA, USA). Statistical significance was defined as follows: * *p* < 0.05, ** *p* < 0.01, and *** *p* < 0.001 for comparisons versus the control group; # *p* < 0.05, ## *p* < 0.01, and ### *p* < 0.001 for comparisons versus the model group.

## 3. Results

### 3.1. Isolation and Bioactivity-Guided Purification of Squid-Derived Antioxidant Peptides

PPSN was fractionated using size-exclusion chromatography on a Sephadex G-25 column(Shanghai Yuanye Bio-Technology Co., Ltd., Shanghai, China). The elution profile revealed three distinct peaks, designated as Fractions I, II, and III. The fractions were collected separately. Following dialysis and lyophilization, the yields were 9.64 mg, 11.13 mg, and 43.89 mg, respectively, based on an initial loading mass of 100 mg PPSN. Notably, the recovery rate of Fraction III (PASN) relative to the initial PPSN was 43.89%.

**Figure 3:**
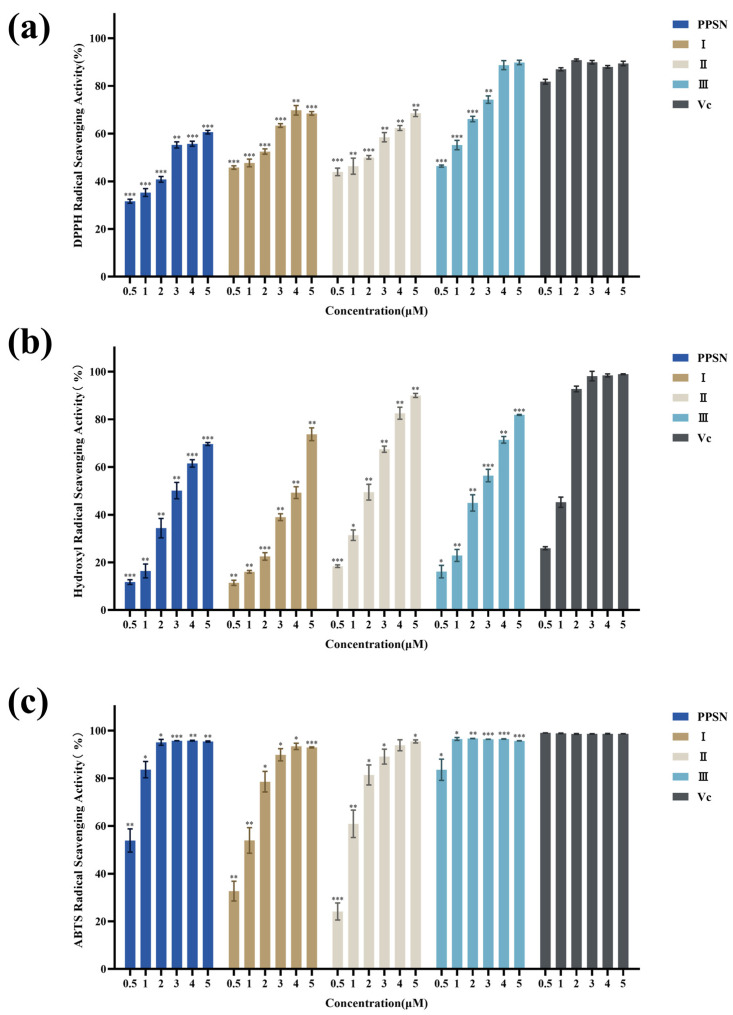

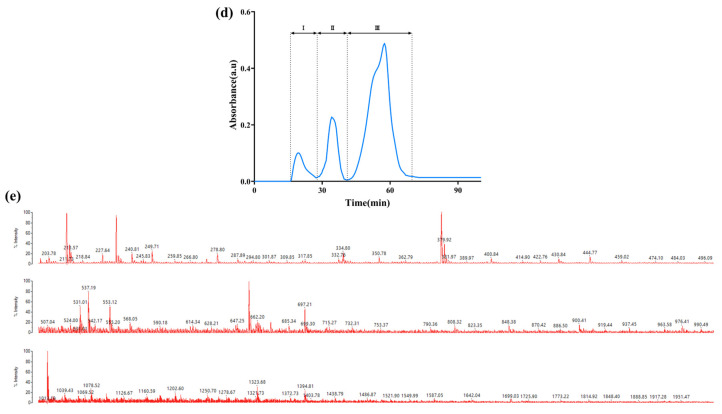
The optimal components were selected as PASN. Elution fractions were subjected to the following assays: DPPH radical scavenging test (**a**), hydroxyl radical scavenging test (**b**), and ABTS radical scavenging test (**c**). Data are presented as mean ± standard deviation (*n* = 3). V_C_ was used as the control. Statistical significance relative to the control group is denoted by * (*p* < 0.05), ** (*p* < 0.01) and *** (*p* < 0.001). Isolate and purify different components from PPSN. Perform dextran gel filtration chromatography on PPSN using a Superdex G-25 column (**d**). PASN QTOF mass spectra in the 200–2000 Da range (**e**). Data are presented as mean ± standard deviation (*n* = 3).

To correlate molecular weight distribution with biological function, the antioxidant capacities of all fractions were assessed using DPPH, ABTS, and hydroxyl radical scavenging assays, with V_C_ serving as the positive control. While all fractions demonstrated radical scavenging activity, Fraction III exhibited significantly higher potency, particularly in the ABTS assay (Figure 3c). In the DPPH assay, Fraction III exhibited an EC_50_ of 0.89 ± 0.06 mg/mL (*p* < 0.001), which was significantly lower than that of PPSN (2.70 ± 0.11 mg/mL, *p* < 0.001) and Fraction II (2.28 ± 0.15 mg/mL, *p* < 0.001). Similarly, Fraction III demonstrated the strongest activity in the ABTS assay, with an EC_50_ of 0.58 ± 0.05 mg/mL (*p* < 0.001), outperforming PPSN (0.72 ± 0.04 mg/mL, *p* < 0.001), Fraction I (1.05 ± 0.08 mg/mL, *p* < 0.001), and Fraction II (0.93 ± 0.07 mg/mL, *p* < 0.01). However, in the hydroxyl radical scavenging assay, the activity of Fraction III (2.68 ± 0.16 mg/mL, *p* < 0.001) was slightly lower than that of Fraction II (2.17 ± 0.14 mg/mL, *p* < 0.001). Although V_C_ exhibited the highest overall activity (EC_50_ < 0.5 mg/mL in the ABTS assay), Fraction III displayed the most balanced and potent antioxidant profile among all the isolated fractions.

Consequently, Fraction III was identified as the lead bioactive component and termed PASN. High-resolution Q-TOF Mass Spectrometry analysis of PASN revealed a primary molecular weight distribution between 211.73 Da and 1013.48 Da (Figure 3e). The prevalence of peaks below 1 kDa confirms that PASN consists predominantly of short-chain oligopeptides rather than intact proteins.

### 3.2. Structural Characterization and Conformational Analysis

**Figure 4:**
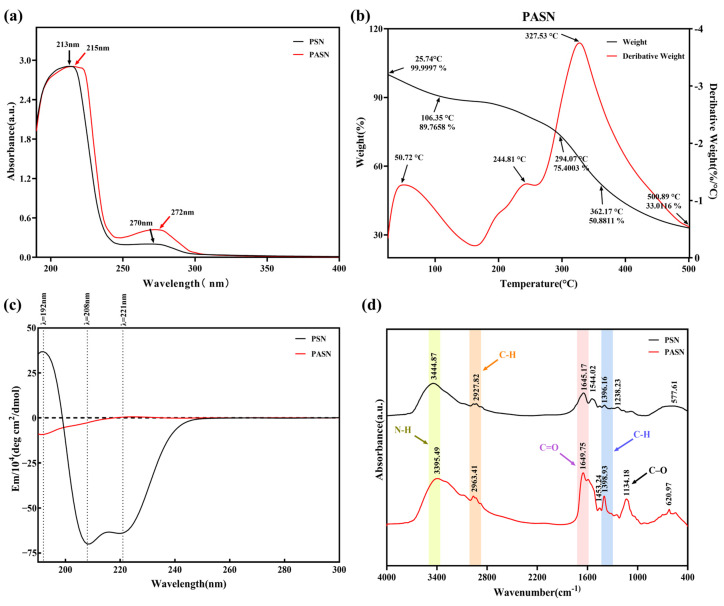
UV-visible spectra of PSN and PASN (**a**), thermogravimetric analysis of PASN (**b**), the CD chromatograms of PSN and PASN (**c**), and FTIR spectra of PSN and PASN (**d**).

The UV-Vis absorption spectra of both PSN and PASN displayed strong maximum absorption peaks in the 200–220 nm range (Figure 4a). This presents the typical UV absorption characteristics of polypeptides, indicating that the samples are peptide substances. TGA that PASN is thermally robust, with moisture desorption occurring below 100 °C and the onset of peptide backbone degradation observed above 240 °C. The main thermal decomposition event peaked at approximately 294 °C (Figure 4b). CD and FTIR spectroscopy were employed to probe the secondary structural transitions during hydrolysis (Figure 4c,d). The CD spectrum of the intact PSN showed a positive cotton effect at 192 nm and double minima at 208 nm and 221 nm, signifying a well-ordered α-helical and β-sheet architecture. In contrast, the PASN spectrum displayed a negative peak at 192 nm, suggesting a loss of long-range order.

**Figure 5:**
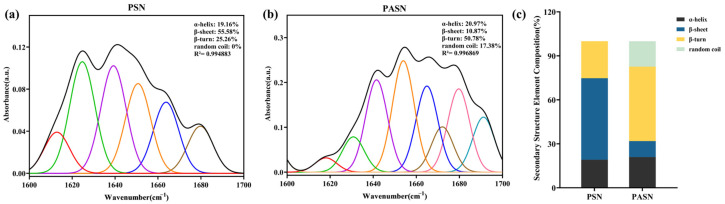
The percentage of each conformation (**c**) was analyzed by Gaussian deconvolution of the amide I region in PSN and PASN (**a**,**b**).

Gaussian deconvolution of the FTIR Amide I band (1600–1700 cm^−1^) provided quantitative insight into these transitions. The intact PSN was dominated by β-sheets (55.58%) and α-helices (19.16%). Following enzymatic cleavage into PASN, there was a notable increase in random coils (17.38%) and β-turns (Figure 5). This transition from a rigid, globular conformation to a flexible, disordered state is consistent with the fragmentation of a large protein into short, bioactive peptides.

### 3.3. Assessment of PASN Cytocompatibility and Proliferative

**Figure 6:**
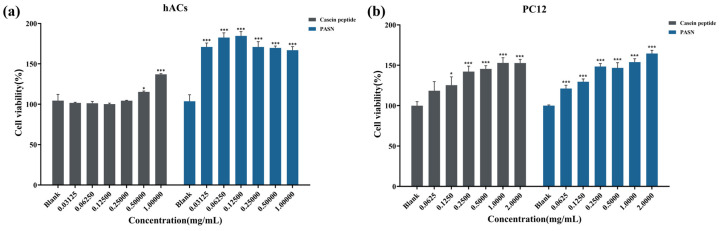
Cytocompatibility of PASN in hACs and PC12 cell lines: (**a**) viability of hACs cells and (**b**) PC12 cells following exposure to varied PASN concentrations (Mean ± SD, *n* = 3). Casein-derived peptide served as a comparative control. Statistical significance relative to the control group is denoted by * (*p* < 0.05) and *** (*p* < 0.001).

To evaluate the safety profile of PASN, cell viability was assessed in PC12 and hACs cells using the CCK-8 assay. As illustrated in Figure 6, PASN exhibited no cytotoxic effects within the tested concentration range, confirming its high biocompatibility. In PC12 cells, PASN treatment elicited a significant, dose-dependent proliferative response (*p* < 0.001), with cell viability peaking at 164.52 ± 3.94% (*p* < 0.001) at the highest concentration tested. Conversely, hACs cells displayed a biphasic response: cell viability increased to 184.56 ± 5.39% (*p* < 0.001) at lower concentrations but showed a relative decline to 166.88 ± 4.43% (*p* < 0.001) at the highest concentrations, though remaining above control levels (*p* < 0.001). These results establish the non-toxic nature of PASN and its potential as a growth-promoting agent.

### 3.4. Neuroprotective Effects of PASN Against Corticosterone-Induced Oxidative

**Figure 7:**
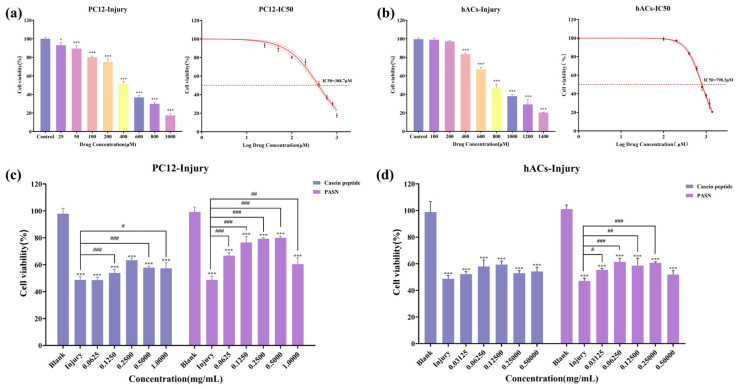
PASN attenuates corticosterone-induced cytotoxicity: (**a**,**b**) Dose–response curves for corticosterone in PC12 and hACs cells to determine IC50. (**c**,**d**) Protective effect of PASN on CORT-treated cells (Mean ± SD, *n* = 3). * and *** indicate *p* < 0.05 and *p* < 0.001 vs. the blank control; #, ##, and ### indicate *p* < 0.05, *p* < 0.01, and *p* < 0.001 vs. the CORT-injured group.

To establish a model of oxidative stress-induced injury, PC12 and hACs cells were treated with varying concentrations of CORT. As shown in (Figure 7a,b), CORT induced a significant reduction in cell viability, with calculated IC_50_ values of 388.7 μM for PC12 and 798.3 μM for hACs. Pre-treatment with PASN significantly attenuated CORT-induced cytotoxicity (Figure 7c,d). For both cell lines, PASN demonstrated a bell-shaped dose–response curve, where survival rates peaked at intermediate concentrations before declining at higher doses. This non-monotonic pattern was statistically validated by the significant difference between the peak response and the response at higher concentrations (e.g., 0.125 mg/mL vs. 1.0 mg/mL, *p* < 0.05). Non-monotonic (bell-shaped) dose–response relationships are well-documented in biological systems, particularly in hormone-related signaling, where adaptive responses often peak at low-to-moderate doses and diminish at higher doses [39,40]. Notably, in hACs cells, concentrations ranging from 0.03125 to 0.25 mg/mL significantly improved survival relative to the CORT-injured group (*p* < 0.05 to *p* < 0.001), suggesting a robust protective window against steroid-induced oxidative damage.

### 3.5. Modulation of LDH Leakage and T-SOD Activity by PASN

**Figure 8:**
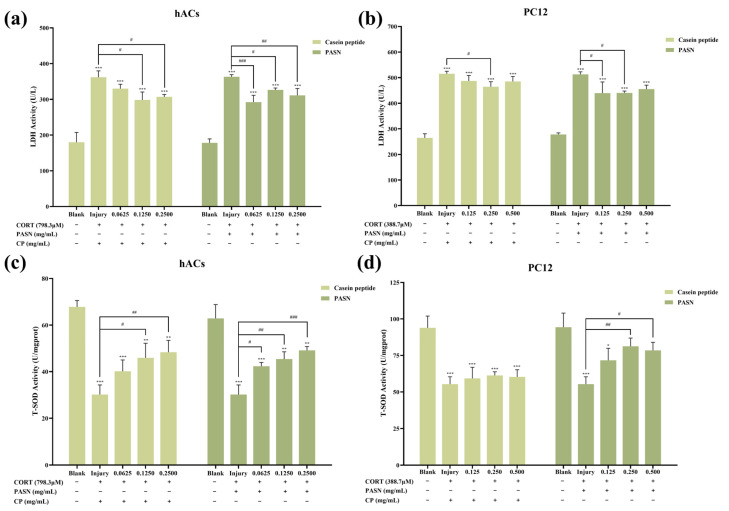
PASN preserves membrane integrity and enhances antioxidant defenses: (**a**,**b**) extracellular LDH activity and (**c**,**d**) intracellular T-SOD activity in hACs and PC12 cells following CORT injury and PASN treatment (Mean ± SD, *n* = 3). Statistical significance relative to the control is marked by *, * *p* < 0.05, ** *p* < 0.01, *** *p* < 0.001; significance relative to the injury group is marked by #, # *p* < 0.05, ## *p* < 0.01, ### *p* < 0.001.

The protective mechanism of PASN was further investigated by measuring LDH release and T-SOD activity. CORT exposure resulted in a sharp increase in extracellular LDH levels to 202.44% in hACs and 189.49% in PC12 cells of the control, indicating compromised plasma membrane integrity (*p* < 0.001, Figure 8a,b). PASN treatment significantly suppressed LDH leakage in a dose-dependent manner, with optimal inhibition observed at 0.25 mg/mL for PC12 (440.31 ± 7.40 U/L, reducing LDH by 14.33%, *p* < 0.05) and 0.0625 mg/mL for hACs (292.34 ± 18.91 U/L, reducing LDH by 19.36%, *p* < 0.001). Concurrently, CORT injury significantly depleted intracellular T-SOD activity to 46.22% in hACs and 58.79% in PC12 cells (*p* < 0.001), reflecting a weakened antioxidant defense system. PASN administration partially restored T-SOD levels in both cell lines, increasing activity to 49.12 ± 1.64 U/mgprot in hACs and 81.21 ± 5.67 U/mgprot in PC12 cells (*p* < 0.001, Figure 8c,d), with the most pronounced enhancement occurring at 0.25 mg/mL (75.18% in hACs and 86.31% in PC12 cells, *p* < 0.05, *p* < 0.001). These findings suggest that PASN protects cells by maintaining membrane structural integrity and augmenting endogenous antioxidant enzyme capacity.

### 3.6. Effect of PASN on BDNF and GAD1/2 Expression

**Figure 9:**
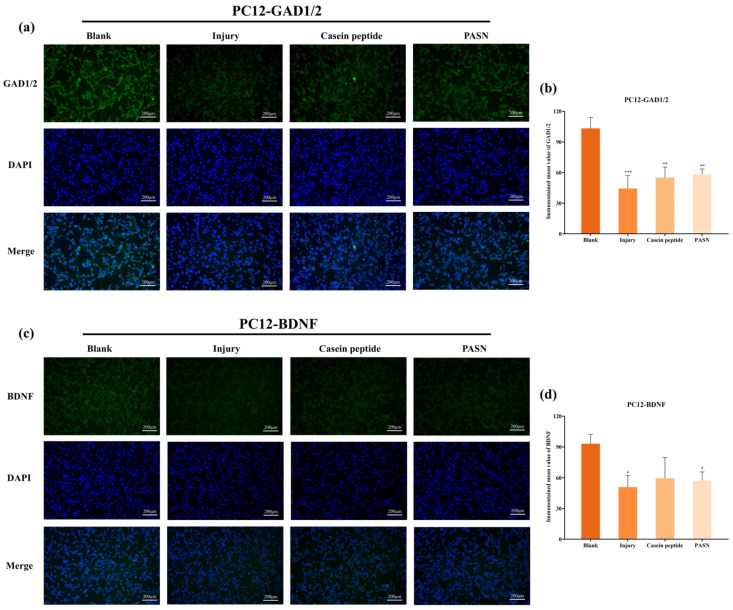
Modulation of BDNF and GAD1/2 in the PC12 injury model: (**a**) representative immunofluorescence images and (**b**) quantitative analysis of GAD1/2; (**c**) IF images and (**d**) quantitative analysis of BDNF expression (Mean ± SD, *n* = 3). Scale bar = 200 μm. * (* *p* < 0.05, ** *p* < 0.01, *** *p* < 0.001) represent comparisons vs. blank groups, respectively.

**Figure 10:**
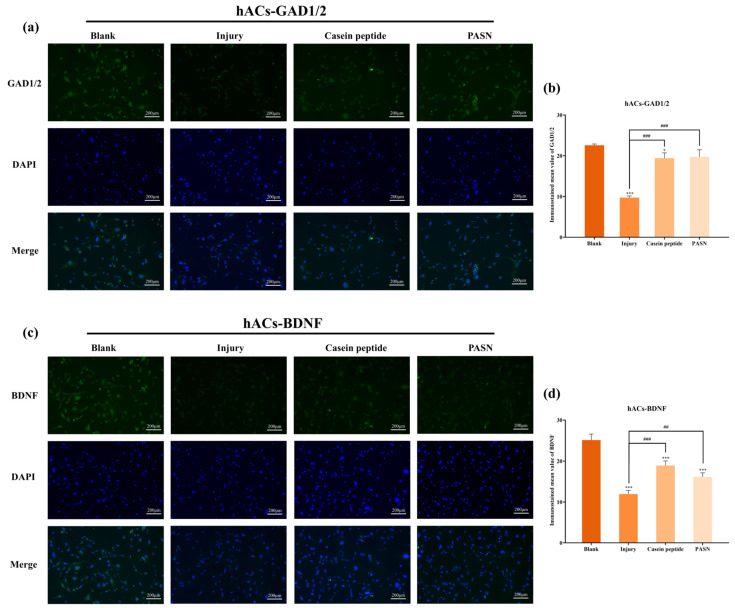
Modulation of BDNF and GAD1/2 in the hACs injury model: (**a**–**d**) representative immunofluorescence and quantitative analysis of GAD1/2 and BDNF expression in hACs cells (Mean ± SD, *n* = 3). Scale bar = 200 μm. Compared with the blank group, * and *** indicate *p* < 0.05 and *p* < 0.001, respectively; compared with the injury group, ## and ### indicate *p* < 0.01 and *p* < 0.001, respectively.

Given the link between stress and neurotrophic depletion, we examined the expression of BDNF and GAD1/2. Immunofluorescence (IF) analysis revealed that CORT-induced injury significantly downregulated the expression of both target proteins (Figure 9 and Figure 10). PASN treatment effectively countered this effect, leading to a partial restoration of BDNF and GAD1/2 fluorescence intensity. These results indicate that PASN may mitigate stress-induced cellular dysfunction by promoting neurotrophic support and stabilizing the GABAergic biosynthetic pathway.

## 4. Discussion

In the present study, a potent antioxidant peptide, PASN, was successfully isolated from Argentine squid protein using Sephadex G-25 gel filtration. The overall yield from carcass byproducts to PASN was 34.28% (dry weight), indicating a feasible valorization route for squid processing waste. Our results demonstrate that PASN is primarily composed of low-molecular-weight species (<1000 Da). This size distribution is a critical determinant of its biological efficacy. LMW peptides are characterized by enhanced transmembrane permeability and superior bioavailability [16,41,42], allowing them to maintain structural integrity under complex physiological conditions consistent with other marine biological peptides [43,44,45,46].

Squid is a seafood species with recognized allergenic potential, and sensitized individuals may react to specific muscle proteins and other allergenic components. Enzymatic hydrolysis is generally considered a practical strategy to reduce allergenicity by disrupting conformational epitopes and generating shorter peptides [47]. However, hydrolysis does not guarantee complete elimination of IgE-binding motifs, and residual allergenic fragments may remain depending on hydrolysis conditions and peptide profiles. Given that PASN is dominated by low-molecular-weight peptides (<1000 Da), the probability of intact allergenic proteins being present is reduced, but dedicated allergenicity assessment is still required. Future work will include (i) in silico screening against curated allergen databases (e.g., IUIS/AllergenOnline) to identify potential sequence homology, (ii) simulated gastrointestinal digestion stability tests, and (iii) in vitro IgE-binding assays using sera from seafood-allergic donors to support a robust safety evaluation before food or pharmaceutical applications [48,49].

The successful isolation of PASN, characterized by a low molecular weight (<1000 Da) and a conformation dominated by β-turns and random coils, distinguishes it from typical marine protein hydrolysates. Unlike the rigid triple-helix structure of its precursor protein, the flexible structure of PASN likely facilitates the exposure of hydrophobic amino acid residues, which are often critical for antioxidant activity [50,51]. This structural transition is not only functionally significant but also confers exceptional thermal stability, with an initial degradation temperature (Td) of 244.81 °C. This property is notably superior to that of many reported marine peptides and collagen derivatives [52]. For instance, while native collagen from cephalopods often exhibits lower thermal stability [53], the enzymatic processing in this study appears to have enriched thermostable fractions. This suggests that PASN possesses a robust structural integrity that can withstand rigorous food processing conditions, such as pasteurization, without significant loss of bioactivity [47], offering a distinct advantage for nutraceutical applications.

The biocompatibility of PASN in both PC12 and hACs cells, coupled with its potent antioxidant capacity, provides a solid foundation for its cytoprotective effects. Our data indicate that PASN operates through a dual mechanism: direct radical scavenging and the fortification of endogenous defenses. While previous studies on marine peptides have primarily focused on their free radical quenching abilities [51,54], our findings reveal that PASN significantly upregulates T-SOD activity. This enzymatic upregulation suggests that PASN triggers an adaptive cellular response, mitigating the cascading effects of CORT-induced oxidative stress more effectively than mere physical scavenging [50]. Consequently, the significant reduction in LDH leakage confirms that PASN preserves membrane integrity, effectively shielding cells from oxidative damage [55,56].

A distinctive and novel contribution of this study is the characterization of PASN’s impact on neuro-regulatory signaling [57,58,59,60]. CORT-induced damage typically precipitates a downregulation of BDNF and GAD1/2, markers intimately linked to synaptic plasticity and sleep–wake regulation [61,62]. Our immunofluorescence data reveal that PASN significantly restores the expression of these proteins. By upregulating BDNF, PASN promotes neuronal survival and enhances synaptic connectivity. More importantly, the restoration of GAD1/2—the rate-limiting enzymes for GABA synthesis—suggests a potential for modulating inhibitory neurotransmission [63,64]. Given that GABA is the primary mediator of sleep induction, these findings indicate that PASN may alleviate sleep disturbances by correcting neurotransmitter imbalances. This focus on the BDNF and GAD distinguishes PASN from other marine peptides, which are often studied solely for general antioxidant properties [65].

In summary, PASN is a thermally stable, biocompatible, environmentally friendly [66], and multifunctional peptide that effectively mitigates CORT-induced oxidative stress and neuro-regulatory dysfunction. Its ability to upregulate BDNF and GAD1/2 positions. Future investigations will focus on full-length sequencing of the peptide components and validating these neuroprotective mechanisms in vitro to facilitate their transition from benchtop discovery to therapeutic application.

## 5. Conclusions

In summary, this study successfully valorized Argentine squid by-products into a novel multifunctional peptide, PASN, through an enzymatic hydrolysis strategy. We demonstrated that PASN possesses a unique structural flexibility and exceptional thermal stability, which underpins its robust antioxidant capacity. Mechanistically, PASN functions as a dual-action agent by not only directly scavenging free radicals but also modulating endogenous cellular defense pathways to mitigate oxidative stress. PASN represents a sustainable, high-value bioactive peptide derived from marine by-products, with outstanding process compatibility and biosafety. It offers a novel natural solution for targeting stress-mediated physiological disorders and supports the green utilization of aquatic processing waste.

Crucially, PASN exhibits a novel neuro-regulatory profile by restoring the expression of BDNF and GAD1/2, key biomarkers for synaptic plasticity and GABAergic signaling. These results underscore the potential of PASN as a high-value, biocompatible candidate for the development of functional foods and pharmaceutical interventions targeting sleep disorders and neurodegenerative pathologies. This work provides a sustainable framework for the valorization of marine by-products into targeted peptide therapeutics.

## Data Availability

The original contributions presented in this study are included in the article/Supplementary Materials. Further inquiries can be directed to the corresponding authors.

## Supplementary Materials

The following supporting information can be downloaded at https://www.mdpi.com/article/10.3390/foods15111844/s1, Table S1: Yield of key processes (using 100 g of Argentine squid mantle by-products as an example); Table S2: Material balance table (using 100 g of Argentine squid mantle by-products as an example).

## Abbreviations

The following abbreviations are used in this manuscript:

CORT	Corticosterone
PSN	Argentine squid protein
PPSN	Argentine squid peptides
PASN	Argentine squid antioxidant peptides
LDH	Lactate dehydrogenase
T-SOD	Total superoxide dismutase
BDNF	Brain-derived neurotrophic factor
GAD1/2	Glutamate decarboxylase 1/2
GABA	γ-aminobutyric acid
hACs	Human astrocytes cells
PC12	Rat adrenal pheochromocytoma cells
UV-vis	Ultraviolet-visible absorption spectrum
CD	Circular dichroism spectra
FTIR	Fourier transform infrared spectroscopy
TGA	Thermogravimetric analysis
