# Supplementary material for: Isolationof PASN from Argentine Squid Carcass By-Products Enhances Proliferation and Repair of hACs and PC12 In Vitro via Antioxidant Activity
Source: Foods. 2026 May 23;15(11):1844. doi: 10.3390/foods15111844 (PMC13256294; doi:10.3390/foods15111844)
Supplement: Supplementary file 1 [file foods-15-01844-s001.zip › foods-4256031-supplementary.pdf]

Preparation of PSN, PPSN, and PASN

1. Extraction of PSN

Pretreatment

Fresh Argentine squid (Dosidicus gigas) were supplied by Zhejiang Zhoushan Haixin Food Co., Ltd. and stored at -20 °C. After thawing, the mantle tissue was retained and cut into 4×4 cm blocks. The blocks were treated in 0.01 mol/L PBS at 4 °C for 8 h with gentle stirring at 30 r/min. The PBS was then discarded, and the samples were immersed in 0.1 mol/L NaOH solution with continuous stirring for 6 h to remove non-protein tissues. The NaOH solution was replaced once during this process. The treated samples were washed three times with ultrapure water, followed by defatting with 8% n-butanol at 4 °C for 12 h. The n-butanol solution was replaced every 4 h. Finally, the tissue was washed repeatedly with ultrapure water until the wash solution reached a neutral pH.

Preparation of Argentine Squid Protein (PSN)

The pretreated mantle tissue was minced into a paste using a chopper with the addition of a small amount of crushed ice. The meat paste was then homogenized using a tissue homogenizer, with a small amount of 0.2 mol/L citric acid solution added to assist the process, ensuring the temperature remained low throughout. The homogenate was mixed with 0.2 mol/L NaOH solution at a solid-to-liquid ratio of 1:4 (w/v) and stirred on a magnetic stirrer for 1 h for extraction. The mixture was centrifuged at 10,000 rpm for 15 min, and the supernatant was collected. This extraction step was repeated 1–2 times.

The combined supernatants were adjusted to the isoelectric point (pH ≈ 5.5) by dropwise addition of 6 mol/L H_2SO_4 under continuous stirring. The precipitate was collected by centrifugation. An appropriate amount of the precipitate was dissolved in 0.5 mol/L glacial acetic acid solution and subjected to ultrasonication for 1 min, followed by centrifugation at 10,000 rpm for 5 min. The resulting supernatant was dialyzed against distilled water at 4 °C using a dialysis bag (MWCO = 200 Da). The dialysis solution (glacial acetic acid) was replaced every 6 h with gradually decreasing concentrations until the solution reached a neutral pH. The dialyzed product was designated as Argentine Squid Protein (PSN).

2. Preparation of PPSN

Selection of Proteases

In this study, Argentine squid protein peptides (PPSN) were prepared using a dual-enzyme hydrolysis method. The candidate proteases included acid protease, neutral protease, alkaline protease, papain, trypsin, and pepsin. To select the optimal proteases for PSN hydrolysis, the hydrolysis reaction was carried out for a fixed duration at the optimal temperature and pH for each specific protease (Table 2-3). PSN was mixed with ultrapure water at a ratio of 1:20 (w/v) for the

hydrolysis reaction. After 4 h of hydrolysis, the enzymes were inactivated by heating for 10 min. The hydrolysate was centrifuged at 5,000 rpm for 10 min. The two most suitable proteases were selected based on the degree of hydrolysis (DH) and peptide concentration.

Optimum reaction conditions of different proteases

Protease	pH	Temperature (°C)
Pepsin	2.5	37
Alkaline protease	10.0	55
Acid protease	3.5	50
Papain	6.0	50
Neutral protease	7.0	50
Trypsin	8.0	37

Single-factor experiments

Optimization of hydrolysis time: Protein solutions with the same concentration were prepared and hydrolyzed for 1, 2, 3, 4, 5, and 6 hours, respectively. While keeping other variables constant, the degree of hydrolysis (DH) and peptide concentration of the hydrolysates were measured.

Optimization of protease ratio: Protein solutions with the same concentration were prepared and hydrolyzed at enzyme ratios of 2:1, 3:2, 1:1, 2:3, and 1:2. The DH and peptide concentration of the hydrolysates were determined.

Optimization of total enzyme dosage: Protein solutions with the same concentration were prepared and hydrolyzed with total enzyme dosages of 1.5%, 2.5%, 3.5%, 4.5%, and 5.5%. The DH and peptide concentration of the hydrolysates were measured.

Optimization of hydrolysis temperature: Protein solutions with the same concentration were prepared and hydrolyzed at temperatures of 40, 45, 50, 55, and 60 °C. The DH and peptide concentration of the hydrolysates were determined.

Optimization of hydrolysis pH: Protein solutions with the same concentration were prepared and hydrolyzed at pH values of 5, 6, 7, 8, and 9. The DH and peptide concentration of the hydrolysates were measured.

Response surface methodology (RSM) optimization of enzymatic hydrolysis conditions

To determine the optimal enzymatic hydrolysis process, three factors—A (hydrolysis pH), B (hydrolysis temperature), and C (total enzyme dosage)—were selected for further optimization based on the results of the single-factor experiments. The factor levels are presented in Table.

Experimental level and factors

Level	A (Hydrolysis pH)	B (Hydrolysis temperature / °C)	C (Total enzyme dosage / %)
-1	6	45	2.5
0	7	50	3.5
1	8	55	4.5

Determination of Degree of Hydrolysis (DH)

The degree of hydrolysis was determined using the formaldehyde titration method. A 5 mL aliquot of the sample solution was diluted to 100 mL with distilled water. Then, 20 mL of the diluted solution was mixed with 100 mL of water and 3 drops of phenolphthalein, and titrated with NaOH solution until a pink color appeared and persisted for 30 seconds. Subsequently, 3 drops of phenolphthalein and 20 mL of formaldehyde were added. After shaking and standing for 1 minute, the solution was titrated again to the endpoint. A blank test was performed by replacing the sample with 100 mL of ultrapure water. To the blank, 3 drops of phenolphthalein and 10 mL of formaldehyde were added, and it was titrated with NaOH solution until a blue-purple color was reached (pH = 9.2).

Determination of Peptide Concentration

The peptide concentration of the samples was determined according to the method described by Lu et al. An equal volume of 5% trichloroacetic acid (TCA) solution was added to the protein peptide solution obtained from enzymatic hydrolysis. The mixture was vortexed thoroughly, allowed to stand for 10 min, and centrifuged at 4,000 rpm for 15 min. The supernatant was collected and diluted 10-fold with 5% TCA solution. The diluted supernatant was then mixed with the biuret reagent at a volume ratio of 3:2 (sample to reagent). After the reaction was complete, the mixture was centrifuged again. The absorbance of the final supernatant was measured at 540 nm, and the peptide concentration was calculated based on a standard curve.

Experimental design and results of response surface

Number	A (enzymolysis pH)	B (Enzymolysis time/h)	C (Total enzyme amount/%)	Y (degree of hydrolysis/%)	Z (Peptide concentration/mg/mL)
1	-1	-1	0		
2	1	-1	0		
3	-1	1	0		

4	1	1	0
5	-1	0	-1
6	1	0	-1
7	-1	0	1
8	1	0	1
9	0	-1	-1
10	0	1	-1
11	0	-1	1
12	0	1	1
13	0	0	0
14	0	0	0
15	0	0	0
16	0	0	0
17	0	0	0

Preparation of PASN

Isolation and Purification

PPSN was purified using Sephadex G-25 (G-25) gel filtration chromatography. The G-25 gel was soaked in distilled water for 24 h to allow for complete swelling. It was then connected to a vacuum pump to remove any trapped gas from the gel for at least 3 h. After degassing, the G-25 slurry was poured uniformly and continuously into the chromatography column to avoid layering and air bubbles. The column was left to stand overnight and then equilibrated with ultrapure water until a stable baseline was achieved. The PPSN was subsequently separated using the G-25 gel column. The eluate corresponding to each peak was collected and dialyzed using a dialysis bag (MWCO = 200 Da) to remove impurities. The purified fractions were stored at -80 °C or preserved by freeze-drying.

Screening of PASN

Based on the results of the DPPH, hydroxyl radical, and ABTS radical scavenging assays, the fraction exhibiting the strongest antioxidant activity and the most favorable qualitative amino acid profile was selected and designated as the Argentine squid antioxidant peptide (PASN).

Table S1. Yield of key processes (using 100 g of Argentine squid mantle by-products as an example)

Name	Weight / g	Yield / %
By-products (Pretreated)	80.87±2.05	80.89±2.08
PSN	13.54±0.62	16.93±0.78
PPSN	9.50±0.37	67.33±2.84
PASN	6.17±0.33	43.89±2.66

Note: The yield is calculated relative to the previous step, following the process: By-products → Pretreated By-products → PSN → PPSN → PASN.

Table S2. Material balance table (using 100 g of Argentine squid mantle by-products as an example)

Consumables	Step	Dosage
Ultrapure water	Washing	7.0 L
	Homogenization	0.4 L
NaOH	Alkaline soaking	4.0 g
	Extraction	3.2 g

DPPH radical scavenging activity assay. 0.1 mmol/L DPPH solution was prepared in anhydrous ethanol. PPSN, purified fractions I–III, and VC were dissolved in ul-trapure water to obtain solutions at concentrations of 1–5 mg/mL. For measurement, 2 mL of DPPH solution was mixed with 2 mL of sample solution and incubated in the dark for 30 min. The absorbance was recorded as A1. The blank control A0 was meas-ured by replacing the sample with anhydrous ethanol. The sample background A2 was determined by replacing DPPH with anhydrous ethanol to eliminate intrinsic absorb-ance of samples. VC was used as the positive control. The scavenging activity was cal-culated as follows: DPPH free radical clearance rate (%) = [1 - (A1 - A2)/A0] × 100%.

Hydroxyl radical scavenging activity assay. Solutions of 0.1 mol/L o-dinitrophenol, 0.1 mol/L $FeSO_4$, 0.01% H_2O_2, and 0.2 mol/L PBS (pH 7.4) were prepared. For measure-ment, 1 mL o-dinitrophenol, 2 mL PBS, and 1 mL $FeSO_4$ were mixed, followed by the addition of 1 mL ultrapure water. The mixture was incubated at 37 °C for 15 min, and the absorbance was recorded as the blank control (A0). The damage control (A1) was determined by replacing ultrapure water with H_2O_2. The sample group (A2) was meas-ured by replacing ultrapure water with the sample solution. VC was used as the posi-tive control. The scavenging activity was calculated as follows: Hydroxyl radical clearance rate (%) = [(A2 - A1)/(A0 - A1)] × 100%.

ABTS radical scavenging activity assay. 7 mmol/L ABTS solution and a 2.45 mmol/L potassium persulfate solution were prepared. The two solutions were mixed in equal volumes to obtain the ABTS stock solution, which was kept in the dark at room temperature for 12–16 h. The stock solution was diluted with anhydrous ethanol to prepare the working solution until the absorbance reached 0.70 ± 0.02 at 734 nm. For measurement, 2 mL of ABTS working solution was mixed with 2 mL of anhydrous ethanol and incubated for 10 min at room temperature; the absorbance was recorded as the blank control (A0). The sample group (A1) was determined by replacing anhy-drous ethanol with the sample solution. The sample background (A2) was measured by replacing ABTS working solution with anhydrous ethanol to eliminate intrinsic inter-ference. VC served as the positive control. The scavenging activity was calculated as follows: ABTS free radical clearance rate (%) = [1 - (A1 - A2)/A0] × 100%.
